# CT694 and pgp3 as Serological Tools for Monitoring Trachoma Programs

**DOI:** 10.1371/journal.pntd.0001873

**Published:** 2012-11-01

**Authors:** E. Brook Goodhew, Jeffrey W. Priest, Delynn M. Moss, Guangming Zhong, Beatriz Munoz, Harran Mkocha, Diana L. Martin, Sheila K. West, Charlotte Gaydos, Patrick J. Lammie

**Affiliations:** 1 Division of Parasitic Diseases and Malaria, Centers for Disease Control and Prevention, Atlanta, Georgia, United States of America; 2 Division of Foodborne, Waterborne, and Environmental Diseases, Centers for Disease Control and Prevention, Atlanta, Georgia, United States of America; 3 Department of Microbiology and Immunology, University of Texas Health Center, San Antonio, Texas, United States of America; 4 Dana Center for Preventive Ophthalmology, Wilmer Eye Institute, Johns Hopkins University, Baltimore, Maryland, United States of America; 5 Kongwa Trachoma Project, Kongwa, Tanzania; 6 Department of Infectious Disease, Johns Hopkins University, Baltimore, Maryland, United States of America; University of California San Diego School of Medicine, United States of America

## Abstract

**Background:**

Defining endpoints for trachoma programs can be a challenge as clinical signs of infection may persist in the absence of detectable bacteria. Antibody-based tests may provide an alternative testing strategy for surveillance during terminal phases of the program. Antibody-based assays, in particular ELISAs, have been shown to be useful to document *C. trachomatis* genital infections, but have not been explored extensively for ocular *C. trachomatis* infections.

**Methodology/Principal Findings:**

An antibody-based multiplex assay was used to test two *C. trachomatis* antigens, pgp3 and CT694, for detection of trachoma antibodies in bloodspots from Tanzanian children (n = 160) collected after multiple rounds of mass azithromycin treatment. Using samples from *C. trachomatis*-positive (by PCR) children from Tanzania (n = 11) and control sera from a non-endemic group of U.S. children (n = 122), IgG responses to both pgp3 and CT694 were determined to be 91% sensitive and 98% specific. Antibody responses of Tanzanian children were analyzed with regard to clinical trachoma, PCR positivity, and age. In general, children with more intense ocular pathology (TF/TI = 2 or most severe) had a higher median antibody response to pgp3 (p = 0.0041) and CT694 (p = 0.0282) than those with normal exams (TF/TI = 0). However, 44% of children with no ocular pathology tested positive for antibody, suggesting prior infection. The median titer of antibody responses for children less than three years of age was significantly lower than those of older children. (p<0.0001 for both antigens).

**Conclusions/Significance:**

The antibody-based multiplex assay is a sensitive and specific additional tool for evaluating trachoma transmission. The assay can also be expanded to include antigens representing different diseases, allowing for a robust assay for monitoring across NTD programs.

## Author Summary

Trachoma is an ocular disease caused by repeated infections with the bacteria *Chlamydia trachomatis* that is observed mostly in children and women. Scarring after repeated infections causes eyelashes to turn under the lid, possibly leading to corneal opacity and blindness. Efforts have increased by multiple organizations to meet the World Health Organization's goal of eliminating trachoma by 2020. As mass drug administration is carried out, it can be difficult to assess whether transmission has lessened enough to stop treatment without resurgence of disease. In this low prevalence setting, sensitive and specific surveillance tools are important. Currently, clinical diagnosis is carried out by examination of the inside of the eyelid for follicles and inflammation, and infection is best assessed using PCR analysis with commercial kits. These test results do not always align, indicating a need for additional tools for surveillance. Using the multiplex assay platform, we compared antibody responses to two chlamydial antigens with eye exams and PCR results of 160 Tanzanian children participating in a mass treatment program. Antibody responses were shown to be a good indicator of infection and disease status and antibody tests may be useful as surveillance tools.

## Introduction

Trachoma, an ocular disease resulting from infection by the bacterium *Chlamydia trachomatis*, causes an estimated 3.8 million cases of blindness and 5.3 million cases of low vision [1] in Africa and Southeast Asia. Trachoma is associated with an estimated $5.3 billion (2003 US dollar calculation) in productivity loss based on impaired vision [2]. Currently, multiple organizations from governments and the private sector are scaling up efforts to eliminate blinding trachoma by 2020 through the World Health Organization's (WHO) Global Elimination of Trachoma by the year 2020 (GET 2020) program. Elimination efforts are based on the components of the SAFE strategy, including Surgery to prevent blindness in those with trichiasis, the use of Antibiotics to treat active infection, the advocacy of Facial hygiene to prevent spread of infection, and Environmental change through sanitation improvements to disrupt transmission. As the cornerstone of the SAFE strategy, Pfizer has donated more than 225 million doses of Zithromax through the International Trachoma Initiative for distribution (www.iti.org).

As neglected tropical disease (NTD) programs reduce infection prevalence, defining program endpoints is a programmatic priority and challenge. This is particularly true for trachoma where clinical pathology may be observed in the absence of active infection [3] or, in some low-prevalence areas, may be caused by inflammation associated with non-Chlamydial bacteria [4]. Monitoring active infection through PCR is an option, but also costly at $10 to $15 per test. The current WHO endpoint for antibiotic use for trachoma is a rate of follicular trachoma less than 5% in children under age 10 years, but clinical exams can be difficult to standardize [5]–[7].

In principle, antibody responses can be used to monitor NTD exposures. Antibody-based tools are being investigated to define their potential contribution to programmatic decision making and surveillance for NTDs including lymphatic filariasis and schistosomiasis [8]–[10]. Antibody assays have been described for chlamydia infections [11], but the utility of these assays for evaluating public health programs has not been extensively investigated. In this study, we used the multiplex bead assay to screen bloodspots from children from four trachoma mesoendemic villages participating in the Partnership for the Rapid Elimination of Trachoma (PRET) Kongwa study [12], [13]. Information to correlate community prevalence (village by village) and individual clinical and laboratory findings (compared with PCR and TF/TI score) was gathered and compared to antibody response to *C. trachomatis* antigens measured by multiplex.

## Materials and Methods

### Study population

Studies were conducted in the Kongwa district (Dodoma region) of Tanzania as part of ongoing clinical trials to evaluate the impact of alternative models of community-wide treatment with azithromycin [12], [13]. As part of routine post-MDA study evaluations, clinical exams, using an expansion of the WHO simplified grading scheme [3], [14] were performed on 100 children several months to 9 years old who were randomly selected from each village, and in four villages all children were examined. Trachoma was graded as zero if the ocular signs did not meet WHO criteria for TF (follicular trachoma) or TI (Trachoma Intense). Grade one TF or TI met the WHO criteria; grade two for TF was if there were 10 or more follicles size >0.5 mm in the tarsal conjunctiva, and TI grade two was present if all the deep tarsal vessels were obscured by inflammation. Eye swabs were collected for PCR analyses of *C. trachomatis* from all children, with careful attention to avoid field contamination. All PCR swabs were shipped to the International Chlamydia laboratory at Johns Hopkins for analyses of infection using Amplicor. Details of laboratory processing are described elsewhere [12]. According to the manufacturer's directions, the Amplicor test was positive if the signal was >0.8 and negative if the signal was <0.2 and equivocal if in-between. All equivocal tests were re-tested, and only counted positive if at least one test was positive.

Four villages were selected for this study because of the schedule for ocular exams post treatment. Of the four villages selected, all received three rounds of treatment. Three villages were 12 months post-treatment (villages 0401, 1602, and 1001) and one was 6 months post-treatment (village 1501). One child per family was selected for collection of bloodspots. Bloodspots were collected following finger prick onto filter papers with six circular extensions designed to absorb 10 µl of whole blood (TropBio Pty Ltd, Townsville, Queensland, Australia). Parents or guardians provided written informed consent for children participating in the study. The study was approved by The Institutional Review Board of the Johns Hopkins University School of Medicine (Baltimore, MD) and the Tanzanian National Institute for Medical Research.

Control samples were included in the analysis. De-linked serum samples from a population of 122 children under the age of 6 years from the United States were collected as part of an IRB approved blood lead study and used as a negative control and to establish a cutoff value for positivity in the multiplex assay. Sera from 86 children under 5 years of age, previously collected from multiple villages outside of Leogane, Haiti as part of IRB approved studies of lymphatic filariasis and other infectious diseases, were also used as a trachoma-negative population as no trachoma has been reported in the country since the 1970's.

### Antigen selection

Candidate antigens were selected based on their recognition by sera from *Chlamydia*-positive patients in published studies [15]. pCT03 (pgp3) is encoded as ORF5 of the eight total ORFs on the highly-conserved cryptic plasmid which is rarely found in *Chlamydia pneumonia* isolates [16]. CT694 is a secreted protein and has been found to be involved in pathogenesis. CT694 manipulates host proteins by acting as a T3S-dependent substrate, but its exact function is not known [17].

### Expression and purification of chlamydial recombinant proteins

CT694 and pgp3 were expressed in the pGEX6p vector system (Amersham Biosciences, Piscataway, NJ) in XL1 Blue. Bacterial cultures were grown to an optical density of 0.7–0.8 in 2× yeast extract and tryptone media with 0.1 mg/ml ampicillin at 37°C. Cultures were induced with 0.2 mM IPTG and incubated three hours at 30°C. Cells were harvested by centrifugation and pellets stored until use at −20°C. All pellets were thawed on ice, suspended in cold PBS with 1 mg/ml chicken egg lysozyme, 1 mM phenylmethylsulfonyl (PMSF), 5 mM EDTA, 0.1 µM Pepstatin A, 0.1 mM *N*-ethylmaleimide, and 0.1 µM E-64. Pellets were sonicated, and then lysis completed using either 1% Tween-20 (pgp3) or 1% Triton-X in PBS (CT694), and finally an additional 0.5 mM PMSF added to all samples. Lysed bacterial cultures were then centrifuged for 20 minutes at 15,000×g to collect the soluble fraction.

The soluble fractions were filtered with a 0.45 micron polypropylene filter (Whatman, Florham Park, NJ) and purified on a glutathione Sepharose 4B affinity column according to the manufacturer's protocol (GE Healthcare, Piscataway, NJ) using PBS buffer. The protein containing glutathione elution fractions were dialyzed overnight (Spectra/Por; 3,500-Da cutoff; Spectrum Laboratories, Rancho Dominguez, CA) against 500 volumes of PBS twice at 4°C. After dialysis, antigen concentrations were quantified by BCA microassay (Pierce, Rockford, IL) and tested for immunologic reactivity by ELISA using previously described methods [18].

### Antigen bead coupling

Antigens were coupled to 5.6 µm polystyrene beads (SeroMap Beads; Luminex Corporation, Austin, TX) as previously described [8]. Briefly, carboxyl groups on the beads were chemically modified to ester groups by 1-ethyl-3-(3-dimethylaminopropyl) carbodiimide (Calbiochem) in the presence of *N*-hydroxysulfosuccinimide (Pierce). Primary amine groups on the antigens then reacted with ester groups on the beads to create amide covalent bonds. Recombinant proteins (120 micrograms each) were coupled with to 1.25×10^7^ beads. Pgp3 and CT694 were coupled in PBS at pH 7.2. After coupling, beads were quantified by hemocytometer and stored at 4°C with protease inhibitors. For each milliliter of bead suspension, 200 µg of Pefabloc (Roche Diagnostics, Indianapolis, IL), 200 µg EDTA, and 1 µg each of leupeptin and pepstatin A were added.

### Bloodspot and sera preparation

One bloodspot extension for each child, corresponding to 10 µl of whole blood, was eluted overnight at 4°C with 500 µl of PBS containing 0.5% BSA, 0.05% Tween 20, 0.02% sodium azide, 0.5% polyvinyl alcohol (PVA), and 0.8% polyvinylpyrrolidone (PVP1), designated as PBN1. This elution was equivalent to a whole blood volume dilution of 1∶50 or a serum dilution of approximately 1∶100. Eluates were diluted (100 µl) to a final volume of 400 µl in PBN1 with 0.5% w/v of *E. coli* crude extract to block nonspecific binding [8]. Sera were diluted 1∶400 in 400 µl PBN1 with 0.5% w/v of *E. coli* crude extract and incubated one hour at 37°C. After incubation, dilutions were stored overnight at 4°C. Sera dilutions were centrifuged at maximum speed to clarify the extract before use.

### Multiplex bead assay

Bloodspot eluates were screened in duplicate with the antigen-coupled beads in a multiplex bead assay [8]. Filter-bottom plates (96-well) (Millipore, Bedford, MA) were pre-wet with 0.5% BSA, 0.05% Tween 20, 0.02% sodium azide in PBS (PBN2). Antigen-coupled beads (2500 each) were added to each well and washed twice with PBN2. Control sera and bloodspot eluates (1∶400) were added in duplicate at 50 µl per well to the beads. The plates were vigorously shaken for 30 seconds, covered, and shaken at room temperature for 1.5 hours. After incubation, wells were washed three times with 100 µl of 0.05% Tween 20 in PBS (PBST) with a vacuum device (Millipore). Total IgG was detected with 50 ng of biotinylated mouse anti-human total IgG (clone H2; Southern Biotech, Birmingham, AL) and 40 ng of biotinylated mouse anti-human IgG4 (clone HP6025; Invitrogen, South San Francisco, CA) per well in 50 µl PBN2. After incubation, wells were washed as above. *R*-phycoerythrin-labeled streptavidin (Invitrogen, South San Francisco, CA) was added at a concentration of 250 ng per well and incubated for 30 minutes at room temperature. Wells were washed as previously after incubation. Wells were additionally incubated in 50 µl of PBN2 to remove any loosely bound antibodies for 30 minutes with shaking. After the final incubation in PBN2, wells were vacuum-evacuated and washed once with PBST. Beads were suspended in 125 µl PBS, shaken, and immediately read on a BioPlex 200 instrument (Bio-Rad, Hercules, CA) equipped with Bio-Plex Manager 6.0 software (Bio-Rad).

### Antigen cross-reactivity tests

Antigens were examined for cross reactivity through two methods: antibody elution from beads and the addition of recombinant protein to block antibodies present in sera. For antibody elution, 150,000 beads of each antigen were suspended in 500 µl PBN1. Sera (2.5 µl) highly responsive to both antigens by ELISA was added to each suspension. The bead mixture was incubated for one hour at room temperature with shaking. Beads were then transferred to a filter-bottom plate (two wells each) and washed six times with 100 µl PBST. Beads were then suspended in 600 µl per well with Gentle Ag/Ab Elution buffer pH 6.6 (Thermo Scientific, Rockford, IL). Bead-containing wells were pooled into a microfuge tube and spun for five minutes at 16,000×g to pellet the beads. Supernatants were then transferred to Centricon-30 centrifugal filter device (Millipore Corporation, Bedford, MA) and 800 µl (0.1 M Tris HCl pH 8.0, 0.3 M NaCl) (THS) added to each sample. Samples were spun at 6000 rpm until concentrated to 50 µl. An additional 2 ml of THS and 4 ml of PBS were added to each and spun to concentrate to 50 µl final volume. Equal volumes of PBN1 were added to each and stored overnight at 4°C. Alternatively, 10 µg of each protein was added to 1∶400 dilutions of sera and incubated for one hour at room temperature. After incubation, serum preparations were stored overnight at 4°C. Sera prepared by both methods were run in the multiplex assay as described above.

### Statistical Analysis

Statistical analysis was conducted using GraphPad Prism 5.03 (GraphPad Software Inc., La Jolla, CA). Specificity and sensitivity were calculated for both antigens by receiver operator characteristic analysis. Comparisons of clinical trachoma, PCR results for infection, and age groups were calculated with confidence intervals (CI) of 95% using Mann-Whitney tests to generate p-values. Villages were compared by Kruskal-Wallis tests with 95% CI.

## Results

### Antigen purification

CT694 and pgp3 antigens were expressed into the bacterial soluble fraction and purified on an affinity column.

### Sensitivity and specificity of antigens

Receiver operator curves (ROC) were generated for each antigen using finger prick sera samples from 122 children from the United States and blood spots from 11 infection PCR positive children from Tanzania. For pgp3, a MFI-BG value of 1024 was established as the low-limit value for positivity, with an indeterminate range of 1024 to 5998. For CT694, a MFI-BG value of 232 was established as the low-limit value for positivity, with an indeterminate range of 232 to 1982.

**Figure 1:**
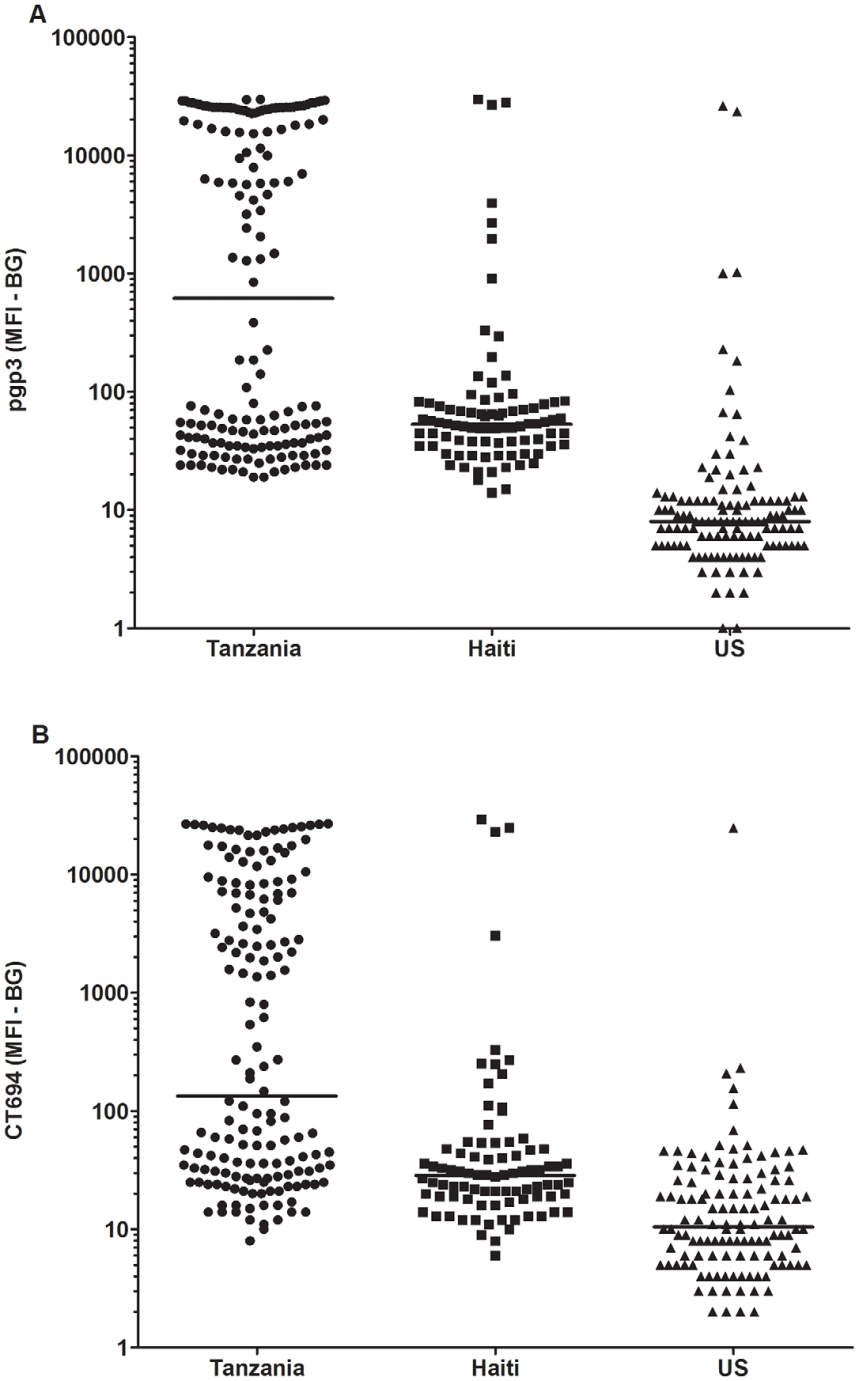
Antibody response to pgp3 and CT694 by country. Median responses are shown in median fluorescence intensity minus background (MFI-BG) by country for (A) pgp3 and (B) CT694. Sera from Haitian children (n = 86) and from United States children (n = 122) were used.

Both antigens detected 10 out of 11 PCR positive children (sensitivity 91%). The PCR positive Tanzanian sample that was not detected in the antibody assay was negative for both pgp3 and CT694. Because PCR data were not available, specificity was determined by using multiplex values of the presumed negative US control children. For pgp3, three presumed negative children were positive (98% specificity) and two children were positive for CT694 (98% specificity). For the US children, only one child was positive for both antigens. Two children were pgp3 positive only and one was positive for CT694 only. Serum samples from 86 Haitian children were also included, representing another trachoma non-endemic country. IgG antibody responses are plotted by country in Figure 1. The median antibody response of Tanzanian children was higher than children from Haiti and the US for both (A) pgp3 and (B) CT694.

### Markers by community

Clinical evaluation results, PCR results from eye swabs, and IgG antibody response to pgp3 and CT694 are shown by village in Table 1. Village 0401 had the highest proportion of children with trachoma and village 1501 had the highest infection by PCR criteria. Village 1001 demonstrated the lowest levels of clinical and antibody positivity. Antibody responses to pgp3 and CT694 were higher than either trachoma or PCR prevalence across all villages.

**Table 1:** Markers by community.

Village	Months post-Tx	n	Trachoma	PCR+	pgp3 Ab+	CT694 Ab+
401	12	36	10 (28%)	4 (11%)	23 (64%)	22 (61%)
1602	12	62	9 (15%)	0 (0%)	33 (53%)	29 (47%)
1001	12	17	1 (5.9%)	1 (5.9%)	4 (24%)	5 (29%)
1501	6	45	4 (8.9%)	6 (13%)	19 (42%)	18 (40%)
Total		160	24 (15%)	11 (6.9%)	79 (49%)	74 (46%)

### Antibody response to pgp3 and CT694

**Figure 2:**
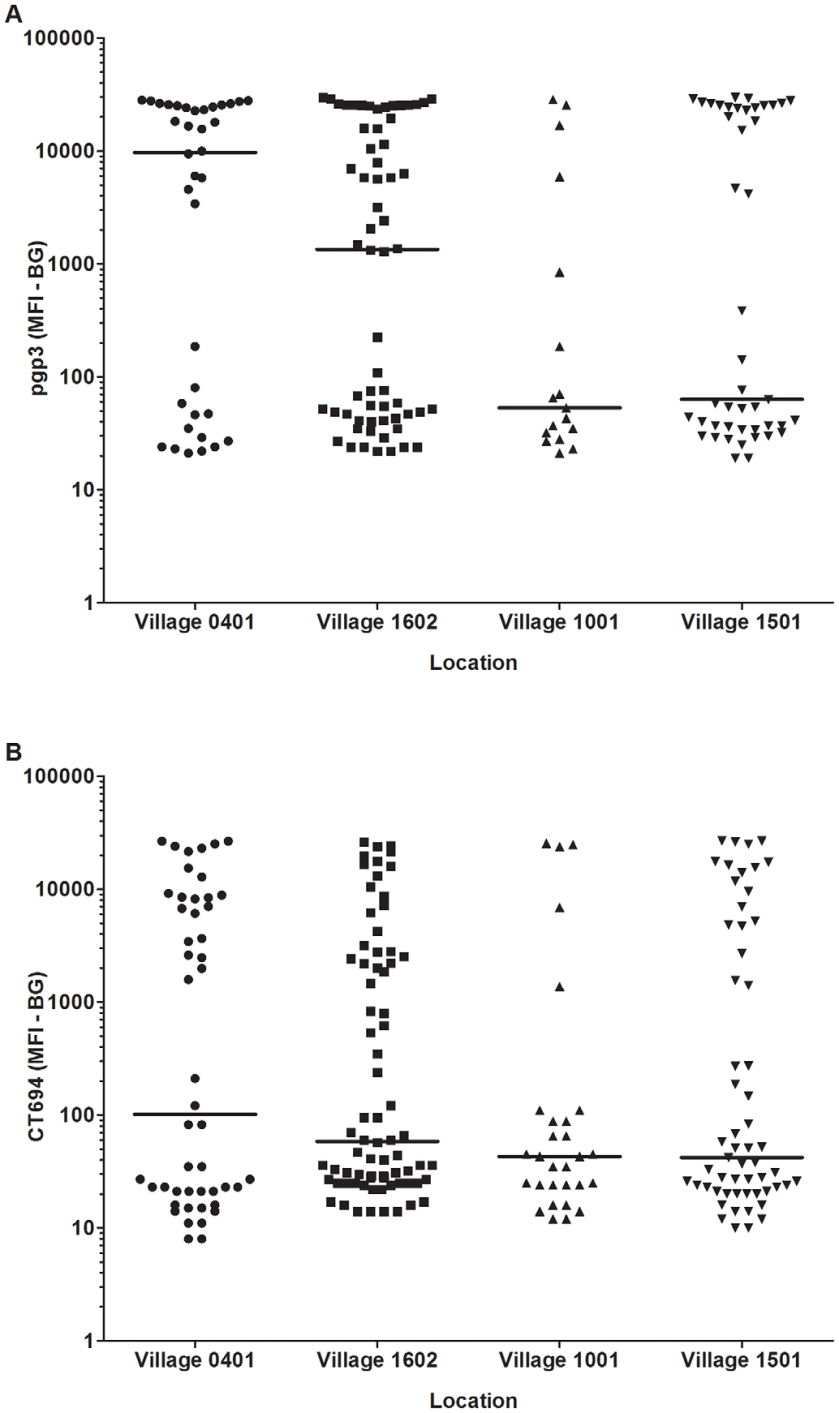
Antibody response to pgp3 and CT694 by village. Median responses are shown in median fluorescence intensity minus background (MFI-BG) by village for (A) pgp3 and (B) CT694.

IgG antibody responses for both recombinant antigens are plotted by village for individual persons in Figure 2. The highest median antibody response was seen in village 0401 for both antigens. The lowest median antibody response was found in village 1001 for both antigens.

**Figure 3:**
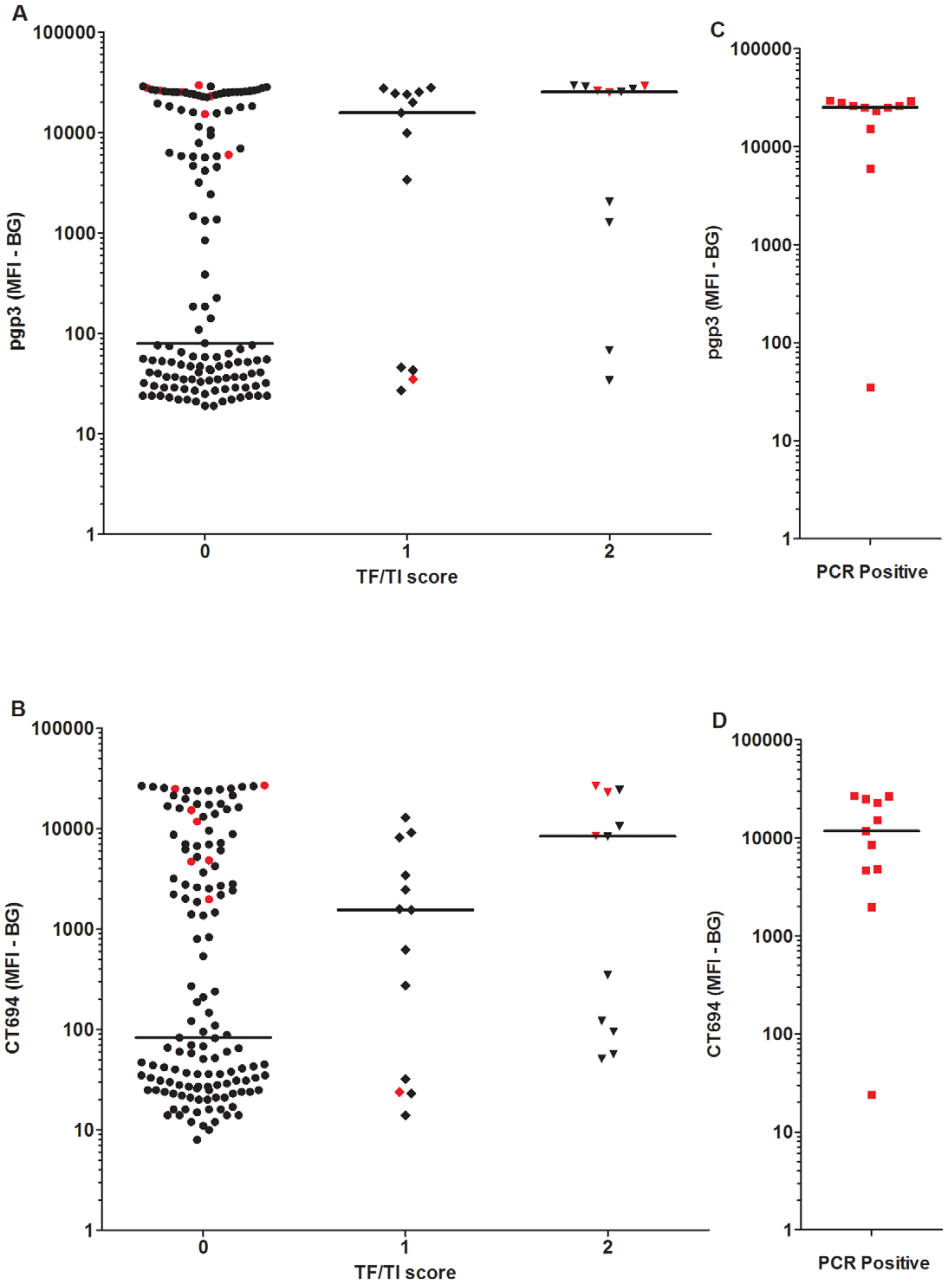
Antibody response by clinical diagnosis and PCR. Median responses are shown in median fluorescence intensity minus background (MFI-BG) for antibody response to (A) pgp3 and (B) CT694 in relation to clinical diagnosis. Antibody response to (C) pgp3 and (D) CT694 is shown by PCR positivity. Responses shown in red indicate PCR positivity. Antibody responses to pgp3 differed between children with no clinical signs and PCR positive children (p = 0.0008) and between children with no clinical signs and those with a TF/TI score of 2 (p = 0.0041). For CT694, antibody responses for children with no clinical signs were lower than for PCR positive children (p = 0.0024) and for those with a TF/TI score of 2 (p = 0.0282).

IgG antibody responses are plotted by trachoma and PCR status for individual persons in Figure 3. Overall, antibody responses to both antigens were associated with ocular pathology and infection status. The median antibody response for children with normal ocular findings (a TF/TI score of zero) is lower than those with either observed ocular disease (pgp3 p = 0.0041 and CT694 p = 0.0282) or PCR positivity (pgp3 p = 0.0008 and CT694 p = 0.0024). All but one PCR-positive individual had a positive antibody response to both pgp3 and CT694. Of note, a large proportion of children with no evidence of ocular pathology had elevated pgp3 and CT694 responses (44% above cutoff for both antigens).

**Figure 4:**
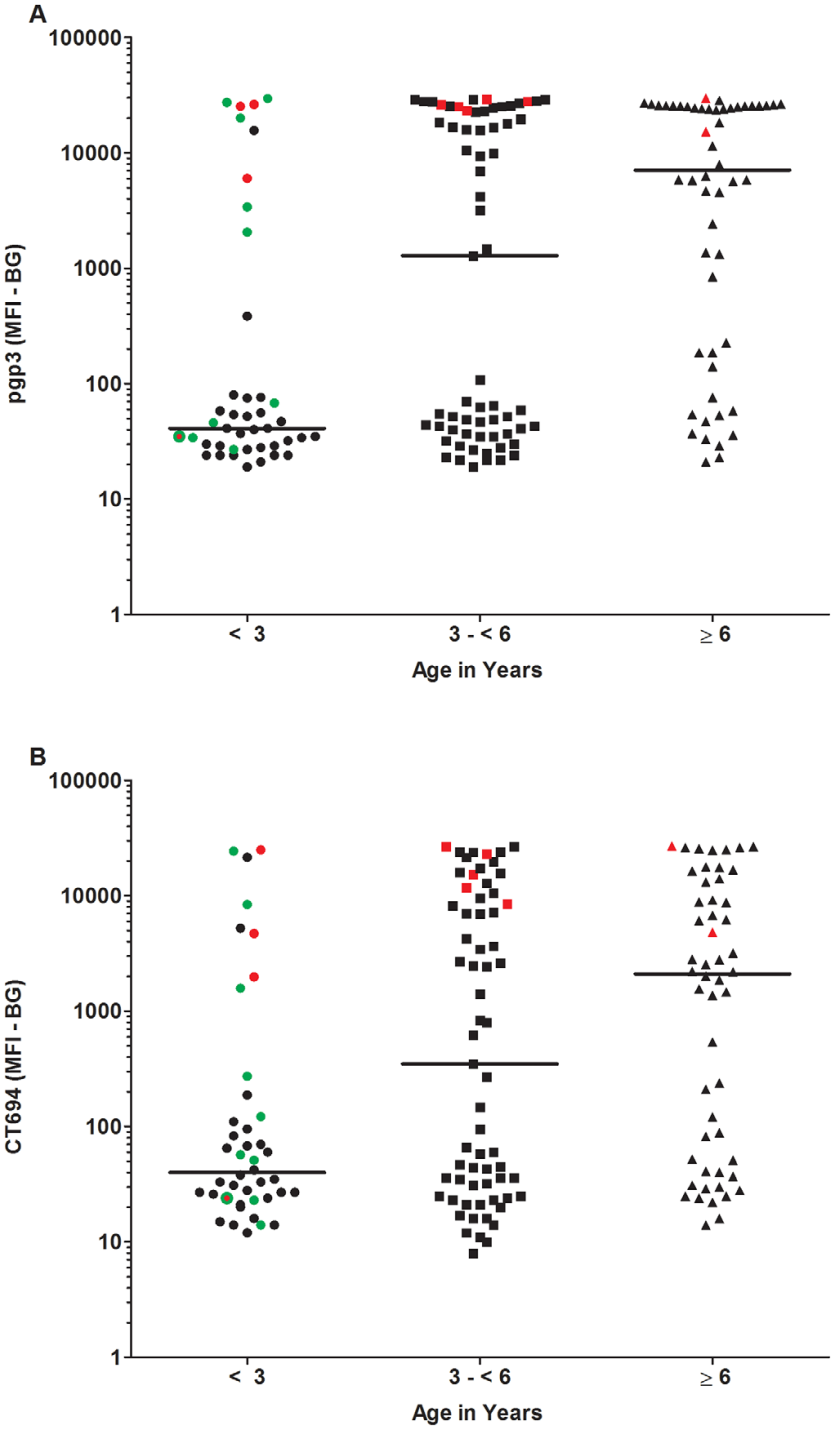
Antibody response in association with age. Median responses are shown in median fluorescence intensity minus background (MFI-BG) for (A) pgp3 and (B) CT694 antibody positive responses by age ranges of less than 3 years old (n = 42), between 3 and less than 6 years old (n = 65), and 6 years or older (n = 52). Responses shown in red indicate PCR positivity. Responses in green are children with ocular pathology (shown only in the <3 age group). Children with both trachoma and PCR positivity are indicated by a red dot with a green border. For pgp3, children younger than three years of age had lower antibody responses than those who were between three and six and those who were older than six years of age (p<0.0001 and p = 0.0062, respectively). For CT694, children younger than three years of age had lower antibody responses than children between three and six (p = 0.0291) and children older than six (p = 0.0001).

In Figure 4A and 4B, pgp3 and CT694 positive antibody responses are shown by age groups. Responses to both antigens increased with age, with the highest proportion of positive children in the 6–9 year old age group. For pgp3, children under three responded significantly less than the other two age groups (p = 0.0062 for 3 to <6 and p<0.0001 for ≥6). For CT694 children under three also responded significantly less than the other age groups (p = 0.0291 for 3 to <6 years and p = 0.0001 for ≥6 years). Interestingly, unlike the other age groups, most of the antibody-positive children under age 3 years had infection (indicated in red) or trachoma (indicated in green), while those with neither were more likely to be antibody negative. This suggests that fewer children in this age group had been previously exposed to infection, as might be expected after three rounds of MDA. The older children, who were in the villages prior to the initiation of MDA, were more likely to be antibody positive in the absence of infection or trachoma, likely indicating past exposure.

### Pgp3 and CT694 cross reactivity

Antigens were tested for cross-reactivity using sera and bloodspots highly responsive to both antigens (n = 6). The addition of recombinant protein reduced detection by multiplex to its corresponding beads by 99.9% (p = 0.0022 for both antigens) and by less than 1% for the other antigen (data not shown). This was true for both sera and bloodspot preparations. One of the high-responding sera was additionally screened by the antibody-elution method and demonstrated similar results. Antibodies eluted from pgp3 beads specifically recognized pgp3 but not CT694-conjugated beads. In the reverse experiment, CT694-specific antibodies recognized CT694 but not pgp3 (data not shown).

## Discussion

Sensitive surveillance tools are important for the determination of exposure levels in low-prevalence settings by program managers who are faced with the decision as to whether or not to stop MDA, often in the absence of PCR data. The development of serological tools to detect antibody responses subsequent to trachoma exposure and infection might provide an alternative to clinical exams and PCR analysis for surveillance as an indicator of interruption of transmission. In principle, children born following MDA should experience fewer infections and this should be reflected by lower antibody responses. The absence of an antibody response to a trachoma antigen might indicate an interruption of transmission in formerly endemic areas. Although trachoma programs have not investigated the potential use of antibody tests for program surveillance, the use of serological markers has been shown to be useful in detecting exposure to *C. trachomatis* in the context of genital infections [19], [20]. In this study, we used serological markers to screen for antibody responses in relation to ocular infections and clinical disease. Two antigens, pgp3 and CT694, were selected for the multiplex assay after an extensive literature search. Pgp3 is the only plasmid-encoded ORF (pORF) secreted into the host cell cytoplasm during infection [15]. Pgp3's function remains unknown but it appears to play a role in pathogenesis. In addition, previous authors have suggested its potential use as a diagnostic marker for genital chlamydial infections [16], [21]. CT694 is expressed during infection as a T3SS effector [17] and has also shown to be recognized by host antibodies [19]. We screened bloodspot eluates from Tanzanian children with two chlamydial antigens to measure antibody responses to trachoma antigens after MDA and compared results to clinical exam and PCR analysis data. We have demonstrated that these antibody responses are related to both disease and infection status, suggesting that responses to these chlamydial antigens should be further explored for utility for trachoma surveillance after MDA.

In our assay, we were able to detect and correlate antibody response with clinical and PCR status both at the community and individual level. At the community level, communities with higher trachoma prevalence also had higher pgp3 and CT694 antibody responses, as seen in Table 1. Village 0401 had the highest levels of pgp3 and CT694 antibody along with the highest numbers of positive clinical exams. Village 1001 showed lowest prevalence of positivity in all three tests. At individual level, we were able to correlate both clinical signs of trachoma and PCR positivity for *Chlamydia* with increased levels of pgp3 and CT694 antibody response. Of children with normal clinical exams, there were many with positive antibody responses. These likely represent children with previous infection [11]. Additional support for this conclusion comes from our analysis of age-specific responses. Antibody-positive children with normal ocular exams were typically older than 3 years of age and, thus, potentially infected prior to the beginning of MDA. In contrast, most antibody-positive children (89% of pgp3 positives and 88% of CT694 positives) younger than three years of age were either PCR-positive or had ocular pathology, and antibody prevalence was significantly lower than among older children.

Based on these results, we suggest that antibody-based tools may be valuable for post-MDA surveillance of trachoma. Lack of antibody in young children may be indicative of interruption of transmission and protection by the SAFE strategy, as shown for responses to pgp3 and CT694 in children less than three years of age. Antibody responses may be especially useful as evaluation tools as they represent a cumulative measure of infection, unlike PCR positivity which may be more transient.

Six children (4%) who showed signs of trachoma through clinical examination exhibited no pgp3 or CT694 antibody. This result may have occurred due to imprecision of clinical diagnosis, in which follicle formation was due not to trachoma but to other causes, such as allergic conjunctivitis. Alternatively, there may be some genetic differences in antibody response at the individual level. Finally, as Ghaem-Maghami et al suggest, there may be some suppression of antibody response in severe cases of inflammation [11]; if this is so, it was rare in our series. Additional studies are needed to define the kinetics of antibody response following infection and how antibody responses shift following repeated infections.

The US samples are unidentified, and, without knowing the child's country of origin and medical and travel history, we do not know their true disease status. There is a possibility that chlamydial infection was acquired at birth [22], [23]. The responses of Haitian children were not as easy to interpret. Eight of 86 children were positive for at least one antigen, and three of these children were positive for both. Even though Haiti is considered non-endemic for trachoma, it is difficult to determine from this sample group if antibody reactivity was due to chlamydial infection acquired at birth (not trachoma) or evidence of acquired chlamydia after birth. It may be that these responses reflect cross reactivity to other antigens. From our own analysis, we have demonstrated that the antibody responses to pgp3 and CT694 do not cross-react.

The multiplex bead assay may be a valuable antibody-based tool for trachoma surveillance. Confirmatory data can be generated by using multiple antigens within the same sample. In theory, antigens might also be selected to distinguish between current infections and exposure or to differentiate infections caused by various serovars. Along with trachoma-specific antigens, antigens for monitoring and evaluation from other NTD public health programs may be included in the multiplex assay. Community profiling can be accomplished through a panel of antigens representing a multitude of diseases, such as helminths, viruses, and waterborne and vaccine-preventable diseases, which can be screened from a single bloodspot or microliter of serum, thus greatly increasing cost-effectiveness of surveillance activities. Using a panel of antigens also facilitates measurement of the impact of MDA on multiple diseases, which might not otherwise be tracked through separate monitoring programs.

There are several limitations to consider in the context of this study. First, all villages have been treated by MDA prior to sample collection, so the baseline antibody responses are unknown for each antigen. Also, because samples were collected at only one time point, our understanding of how each child's antibody and disease status changed over time is limited. These points make it difficult to describe the kinetics and longevity of antibody responses with the antigens tested and, more specifically, whether antibody responses reflect current or previous infection. A longitudinal study, including a baseline collection is an important next step to confirm the value of the antibody tools described here. Because antigens were chosen based on literature specific to genital chlamydial infections [15], we cannot be sure that these antigen choices are the most appropriate in terms of ocular infection. For example, cases of anti-chlamydial responses but no ocular pathology may be due to a non-ocular strain of *Chlamydia*. The use of serovar-specific antigens, such as the major outer membrane protein peptides, might distinguish between genital and ocular infections and better characterize the nature of the infecting bacteria.

In summary, antibody-based assays, in particular the multiplex assay, could be valuable tools to evaluate the impact of MDA programs and for post-MDA surveillance for trachoma.
